# Systematic levels of IL-29 and microRNA185-5p were not associated with severe COVID-19 in the Iranian population

**DOI:** 10.1186/s12985-023-02046-7

**Published:** 2023-05-05

**Authors:** Omidreza Sarrafi, Ashraf Kariminik, Mohammad Kazemi Arababadi

**Affiliations:** 1 Department of Microbiology, Kerman Branch, Islamic Azad University, Kerman, Iran; 2 Immunology of Infectious Diseases Research Center, Research Institute of Basic Medical Sciences, Rafsanjan University of Medical Sciences, Rafsanjan, Iran; 3 Department of Laboratory Sciences, Faculty of Paramedicine, Rafsanjan University of Medical Sciences, Rafsanjan, Iran

**Keywords:** COVID-19, IL-29, microRNA185-5p

## Abstract

**Background:**

Increased systematic pro-inflammatory cytokines is the main cause of the inflammatory conditions of the hospitalized severe acute respiratory syndrome coronavirus 2 (SARS-CoV-2) infected patients. In this project, serum levels of IL-29 and whole blood levels of microRNA-185-5p (miR-185-5p) were evaluated in the hospitalized SARS-CoV-2 infected patients.

**Methods:**

This project was performed on the 60 hospitalized SARS-CoV-2 infected patients and 60 healthy controls to evaluate IL-29 and miR185-5p expression levels. IL-29 expression was explored using enzyme linked immunoassay (ELISA), while miR185-5p was evaluated using Real-Time PCR techniques.

**Results:**

The results demonstrated that neither IL-29 serum levels nor relative expressions of miR-185-5p were significantly different between patients and healthy controls.

**Conclusion:**

Due to the results that are presented here, systematic levels of IL-29 and miR-185-5p cannot be considered as the main risk factors for induction of inflammation in the hospitalized SARS-CoV-2 infected patients.

**Supplementary Information:**

The online version contains supplementary material available at 10.1186/s12985-023-02046-7.

## Background

The current severe acute respiratory syndrome coronavirus 2 (SARS-CoV-2) pandemic (COVID-19) is associated with involvement of several tissues, including lung and heart, via over-activation of cellular immunity [1, 2]. The roles played by the pro-inflammatory cytokines in the pathogenesis of COVID-19 have been demonstrated in several investigations [3, 4]. However, the various ethnics show different patterns of the pro-inflammatory molecules, demonstrating the genetic and epigenetic factors play crucial roles in determining gene expressions [3, 4]. Additionally, understanding of the main mechanisms result in severe symptoms in SARS-CoV-2 infected patients, can promote designing some therapeutic strategies. Interleukin (IL)-29 belongs to type III interferons and plays important roles in the immune response against viruses by inducing the mechanisms similar to type I interferons [5]. Accordingly, the cytokine activates expressions of several molecules, including interferon-stimulated genes and antiviral proteins [6]. Based on the anti-viral activities of IL-29, it has been documented that the cytokine is an important factor against respiratory viral infections [7]. Therefore, it may be a part of immune responses against SARS-CoV-2 and the related cytokine storms.

As mentioned above, several genetic and epigenetic factors can regulate expression of immune responses-related genes [8]. MicroRNAs (miRs), as the epigenetic factors, are the essential factors participate in the regulation of translation via interactions with the target mRNA [9]. It has been reported that miR185-5p is the key regulators of cellular immunity [10, 11]. Additionally, the significant roles played by miR185-5p in the regulation of immune responses have been documented previously [12]. Due to the fact that both IL-29 and miR185-5p play key roles in regulation of immune responses against viral infections, it appears that the molecules may participate in the pathogenesis of COVID-19. Additionally, there were no investigations to explore the molecules in the patients suffering from severe COVID-19 in Iranian patients, hence, this project was designed to explore the mechanisms used by the molecules to induce inflammation in the Iranian patients. In another word, this project was aimed to evaluate IL-29 and miR185-5p levels in the COVID-19 infected patients who suffer from pro-inflammatory reactions and were hospitalized in the CCU department.

## Methods

### Subjects

In this project, 80 healthy controls and eighty hospitalized SARS-CoV-2 infected patients were included at the first point. Fifteen patients were excluded due to exclusion criteria, three patients were excluded due to missing information, and two patients were excluded due to death. Finally, 60 healthy controls (20 men and 40 women) and 60 hospitalized SARS-CoV-2 infected patients (28 men and 32 women) were explored regarding the expression of miR185-5p and serum levels of IL-29. Due to the more hospitalized women patients, the participants were female. The patients were selected from hospitalized patients in the Afzalipour Hospital, Kerman University of Medical Sciences, Kerman, Iran, randomly. To confirm the infections of SARS-CoV-2 infections, the quantitative PCR test was used. The patients had the clinical symptoms of the severe disease, including respiratory distress (over 20 breaths per minute), more than 50% lung involvement, blood oxygen levels less than 90%, and need for intubation [13]. The patients with smoking, other pathogen infections, hypersensitivities, and consuming opium/immune suppressor drugs were excluded from the study. Before hospitalization and treatment, the informed consent form was completed by the patients and the blood samples were collected in a pre-treated anticoagulant agent tube for extraction of microRNAs and without anticoagulant tubes for separation of serum to evaluate IL-29 serum levels.

### Real-time PCR for detection of SARS-CoV-2

To detect SARS-CoV-2 infection, the viral RNA was extracted using a commercial kit (Karmania Pars Gene Company, Kerman, Iran). The extracted viral-RNA was converted to cDNA and then detected by specific primers and TaqMan probe in FAM canal, using a high-quality one-step SARS-CoV-2 Real-Time PCR kit (Karmania Pars Gene Company, Kerman, Iran). RNase P was detected in the HEX canal simultaneously as the internal control for Real-Time PCR.

### Evaluation of IL-29 serum levels

IL-29 was evaluated in the serum using commercial kits from Karmania Pars gene Company, Kerman, Iran and based on the kit guideline.

### MicroRNA extraction and detection of miR185-5p

MicroRNAs were extracted from whole blood samples using a commercial kit (Karmania Pars Gene Company, Kerman, Iran). MiR-185 were converted to cDNA using a specific cDNA synthesize kit and detected by a CYBR Green-based Real-Time PCR kit (Karmania Pars Gene Company, Kerman, Iran) in a Rotor-Gene Q thermal cycler (Qiagen, USA). The following program was run to amplify miR185-5p in parallel with U6, as the housekeeping gene: 95ºC for 3 min for 1 cycle, 95ºC, 58ºC, and 62ºC for 10, 30, and 30 s, respectively. The melt curve program was run to evaluate the quality of the amplifications and 2^− ΔΔCt^ formula were used to calculate the results.

### Statistical analysis

Kolmogorov Smirnov test (SPSS software version 16) revealed normal data distribution, hence the differences regarding miR185-5p levels of IL-29 serum levels between the groups were calculated using independent student t test. To analyze the correlations between serum levels of IL-29 and relative expression of miR185-5p, the Pearson correlation test was used.

## Results

Statistical analysis revealed that the groups were matched regarding age (*P* = 0.826) and sex (*P* = 0.122). Accordingly, the mean age of the patients was 50 ± 10 and the controls were 48 ± 12 years old. Ten out of 60 patients had a license, 15 patients had a diploma and 35 patients had under diploma education. While it was 8, 17, and 35, respectively, for controls.

**Fig. 1:**
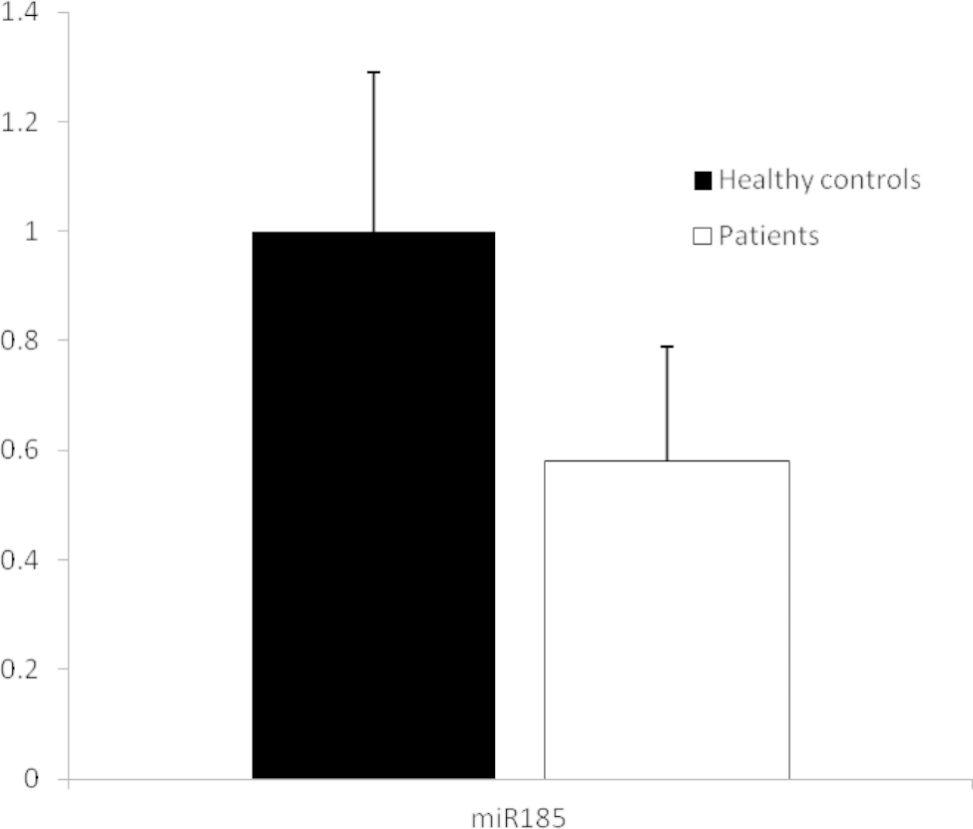
Relative expression of miR185 in the hospitalized SARS-CoV-2 infected patients and healthy controls. The results showed that miR185 (*P* = 0.318) expressions were not changed in the patients when compared to healthy controls

The results demonstrated that the mean relative expressions of miR185-5p were 0.58 ± 0.21 and 1 ± 0.29 in the hospitalized SARS-CoV-2 infected patients and healthy controls, respectively, which were not significantly different (*P* = 0.318, Fig. 1).

**Fig. 2:**
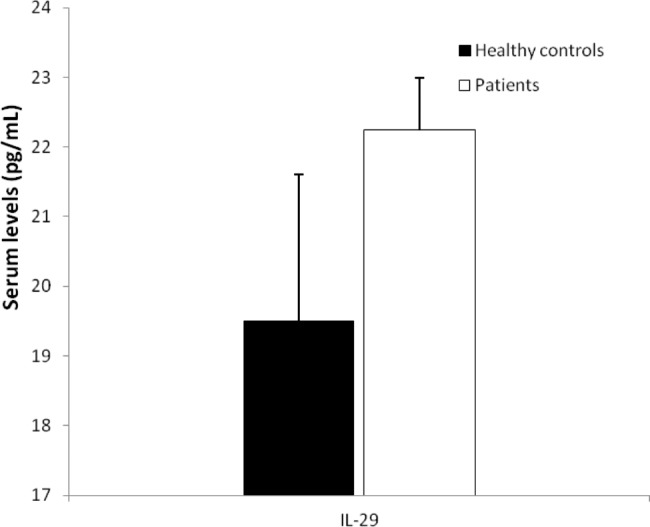
IL-29 serum levels in the hospitalized SARS-CoV-2 infected patients and healthy controls. Serum levels of IL-29 (*P* = 0.146) were not significantly altered in the patients in comparison to healthy controls

Statistical analysis revealed that serum levels of IL-29 were not changed (*P* = 0.146) between hospitalized SARS-CoV-2 infected patients (22.25 ± 0.74 pg/mL) in comparison to healthy controls (19.51 ± 2.09 pg/mL). Figure 2 shows the IL-29 serum levels in the patients and controls.

Independent t test revealed that men patients (0.26 ± 0.12) had significantly decreased miR185-5p levels when compared to the women patients (1.11 ± 0.28 *P* = 0.017), while IL-29 levels were not changed between men and women (*P* = 0.760) in the hospitalized SARS-CoV-2 infected patients.

Pearson test revealed that the relative expression of miR185-5p had not significant correlation with IL-29 serum levels in the patients (Rs = 0.173, *P* = 0.370).

## Discussion

The results revealed that the serum levels of IL-29 were not different between patients and controls. Due to the fact that the hospitalized SARS-CoV-2 infected patients suffer from pro-inflammatory reactions, and based on the fact that IL-29, as a pro-inflammatory cytokine, did not alter in the patients, hence it may be concluded that inflammation in the hospitalized SARS-CoV-2 infected patients is independent of IL-29. However, based on the fact that a main source of IL-29 is the epithelial cells [14], and SARS-CoV-2 infected the cells, so it may be hypothesized that local production of IL-29 participates in the defense against SARS-CoV-2, which is not associated with its elevated serum levels. Thus, it seems that evaluation of the local levels of IL-29 can be useful to understand the roles played by IL-29 against SARS-CoV-2 and its pathogenesis. In parallel with our results, Fallah Vastani et al., reported that serum levels of IL-29 were not changed between the SARS-CoV-2 infected patients with mild and severe symptoms [15]. The results demonstrated that IL-29 does not participate in the induction of inflammation in the SARS-CoV-2 patients. However, they reported that IL-29 levels were significantly higher in recovered patients when compared to the dead patients [15]. The protective roles played by IL-29 against viral infection of respiratory epithelial cells have been reported by Wang and colleagues [16]. Accordingly, they demonstrated interaction of IL-29 with alveolar type II epithelial cells leads to induction of antiviral genes, such as IFN-stimulated gene 56 (ISG56), myxovirus resistance protein 1,2’-5’-oligoadenylate synthetase 1 [16]. Thus, it appears that IL-29 may be considered as an important local molecule against SARS-CoV-2. In agreement with our hypothesis, a review article by Portela Sousa and colleagues revealed that IL-29 participates in protection of epithelial surface barriers against viral infections without generating systemic immune system activation [17]. Our results also demonstrated that IL-29 did not increase systematically in the hospitalized SARS-CoV-2 infected patients.

The results also demonstrated that miR185-5p was decreased in the patients when compared to healthy controls, but it was not statistically significant. A study by Martínez-Fleta et al., demonstrated that plasma levels of miR185-5p were decreased in the severe in comparison to the mild patients [18]. Nicoletti and colleagues also revealed that serum levels of miR185-5p significantly decreased in the SARS-CoV-2 infected patients with severe when compared to the mild symptoms [19]. Additionally, it has been documented that miR185-5p plays anti-inflammatory roles in the resident macrophages [12]. Our results also demonstrated that male patients had lower levels of miR185-5p than females. Thus, gender may be considered as an important factor for expression of the miR. However, Grehl et al., reported that serum levels of miR185-5p were significantly increased in the severe COVID-9 patients when compared to the mild patients [20]. Due to the controversy, it appears that more investigations regarding the roles played by miR185-5p in COVID-19 need to be done to clear the main pro-inflammatory mechanisms.

The strengths of this study were the novelty in Iranian patients and evaluation of protein levels of IL-29, rather than mRNA, while the limitation of this study was the low sample size.

## Conclusion

Due to the results, it may be hypothesized that IL-29 cannot participate in the induction of systemic inflammation in the COVID-19 patients and the roles played by the cytokine and miR185-5p needs to be explored locally in the epithelial surface barriers. However, it appears that the results of this study help physicians to consider IL-29 as a main target to regulate severe inflammation in the hospitalized COVID-19 patients.

## Electronic supplementary material

Below is the link to the electronic supplementary material.

Supplementary Material 1

Supplementary Material 2

Supplementary Material 3

## Data Availability

Data and materials are available.

## Data Availability

Data and materials are available.
