# Supplementary material for: Systematic levels of IL-29 and microRNA185-5p were not associated with severe COVID-19 in the Iranian population
Source: Virol J. 2023 May 5;20:88. doi: 10.1186/s12985-023-02046-7 (PMC10160707; doi:10.1186/s12985-023-02046-7)
Supplement: Supplementary file 1 — Supplementary Material 1 [file 12985_2023_2046_MOESM1_ESM.docx]

Statistical analysis

Evaluation of miR185-5p by Real-Time PCR

Specific cDNA synthesizes

Evaluation of IL-29 by ELISA

Micro-RNA extraction

Serum

WBC

Blood sampling

Selection of the patients and healthy controls
