# Supplementary material for: Systematic levels of IL-29 and microRNA185-5p were not associated with severe COVID-19 in the Iranian population
Source: Virol J. 2023 May 5;20:88. doi: 10.1186/s12985-023-02046-7 (PMC10160707; doi:10.1186/s12985-023-02046-7)
Supplement: Supplementary file 2 — Supplementary Material 2 [file 12985_2023_2046_MOESM2_ESM.docx]

**Sixty** patients and equal number of controls were included at the end point

**Fifteen** patients were excluded due to exclusion criteria

**Three** patients were excluded due to missing information

**Two** patients were excluded due to death

**Eighty** heathy controls and **eighty** patients were included at the first point
